# First report of novel single nucleotide polymorphisms (SNPs) and genetic characteristics of the prion-like protein gene (*PRND*) in cynomolgus monkeys

**DOI:** 10.3389/fvets.2026.1946289

**Published:** 2026-09-30

**Authors:** Chung-Bae Oh, Da-In Choi, Byung-Hoon Jeong

**Affiliations:** 1 Korea Zoonosis Research Institute, Jeonbuk National University, Iksan, Republic of Korea; 2 Department of Bioactive Material Sciences and Institute for Molecular Biology and Genetics, Jeonbuk National University, Jeonju, Republic of Korea; 3 Korea Institute of Toxicology, Jeongeup, Jeonbuk, Republic of Korea

**Keywords:** cynomolgus monkeys, Doppel, prion diseases, *PRND*, snps

## Abstract

Transmissible spongiform encephalopathies (TSEs) are fatal neurodegenerative diseases caused by misfolded prion protein. The prion-like protein (Doppel), encoded by the prion-like protein gene (*PRND*), is linked to neurodegeneration and TSE susceptibility. While *PRND* polymorphisms have been studied in various species, their genetic features in non-human primates remain unexplored. Cynomolgus monkeys (*Macaca fascicularis*), which are genetically close to humans, are a valuable model to study the role of *PRND* polymorphisms in prion disease susceptibility. We performed polymerase chain reaction (PCR) to amplify the monkey *PRND* gene and investigated genetic polymorphisms in cynomolgus monkeys (*n* = 121). Genotype, allele, and haplotype frequencies, as well as linkage disequilibrium (LD), were analyzed to assess the genetic architecture of the population using Haploview (v4.2). Monkey *PRND* amino acid sequences were compared with those of several species using ClustalW. The effects of non-synonymous SNPs and the structural analysis of monkey Doppel were evaluated using *in silico* tools. We identified 15 novel genetic polymorphisms in the monkey *PRND* gene, including 12 variants located within the open reading frame (ORF), one in the upstream region, and two in the downstream region. In addition, LD analysis revealed several strong LD relationships among *PRND* polymorphisms in monkeys. ClustalW analysis showed that humans and monkeys shared the same *PRND* sequence length and exhibited high sequence homology. *In silico* analyses demonstrated that the non-synonymous SNPs, c.61C>T (L21F) and c.370G>C (D124H), were predicted to have damaging effects. This is the first report on the genetic and structural features of *PRND* polymorphisms in monkeys, showing that several non-synonymous amino acid substitutions may affect the structural stability of monkey Doppel proteins.

## 1 Introduction

Prions are unconventional infectious agents responsible for a group of invariably fatal neurodegenerative disorders across diverse species, including humans, and are pathologically defined by spongiform degeneration within the central nervous system (CNS) (1). The prion protein (PrP), a host-derived glycoprotein, is anchored mainly to neuronal surfaces and, to a lesser extent, to lymphocytes and additional cell populations (2, 3). The transmissible agent underlying scrapie is widely accepted to consist of a conformationally altered isoform of the prion protein (PrP) (4). In the course of investigating prion protein gene (*PRNP*)-knockout mice, which initially displayed no overt phenotypic abnormalities, the gene encoding the prion-like protein (Doppel) was unexpectedly identified. Subsequent analyses revealed that Doppel shares structural similarities with PrP, yet its ectopic expression in the CNS is associated with late-onset neurodegeneration. These findings not only provided critical insight into the functional redundancy and divergence between PrP and Doppel but also underscored the complexity of prion biology and the necessity of dissecting host factors that modulate susceptibility to prion pathogenesis.

The prion-like protein gene (*PRND*) is approximately located 16–52 kb downstream of the prion protein gene (*PRNP*) locus (5, 6). Doppel has been described as a product of evolutionary specialization. Doppel exhibits structural similarity to PrP but lacks the characteristic octapeptide repeat region (7). Doppel fails to substitute for the function of PrP and instead exerts a detrimental effect on neuronal survival, indicating that *PRND* is an evolutionarily specialized gene that, despite its structural similarity to PrP, displays distinct functional properties. Their modes of Cu^2+^ coordination and functional consequences diverge markedly (8). Doppel mRNA is expressed during embryogenesis, shows low levels in the adult brain, and is highly expressed in the testis (9). This divergence underscores the functional heterogeneity within the prion protein family and suggests that Doppel may fulfill physiological roles distinct from the pathogenic mechanisms underlying prion diseases.

Numerous case-control studies across diverse mammalian species have associated *PRND* polymorphisms with altered susceptibility and phenotypic variation in prion diseases. In humans, specific polymorphisms, including the 3' UTR +28 variant, T26M, P56L, and T174M, have been associated with the risk and phenotypic expression of Creutzfeldt–Jakob disease (CJD) (10, 11). In addition, *PRND* polymorphisms have been considered important genetic factors in determining susceptibility to prion disease and behavioral disturbances in Alzheimer's disease (12). Moreover, recent studies have demonstrated strong linkage disequilibrium (LD) between *PRNP* and *PRND* polymorphisms in prion disease–susceptible species, including sheep and goats (13, 14). Despite these findings, the genetic characteristics of *PRND* have not yet been investigated in cynomolgus monkeys (*Macaca fascicularis*). Therefore, a comprehensive analysis of *PRND* genetic variation in monkeys represents a critical baseline study that may provide important insights into the molecular mechanisms underlying prion disease susceptibility and resistance.

Although the functional significance of genetic polymorphisms deserves additional exploration, they serve as a framework for understanding genetic vulnerability in primates. Comparative investigations between humans and monkeys have revealed both similarities and differences in *PRND* sequences and prion disease characteristics. Such research is critical for validating monkey models of prion disorders and translating findings to human prion disorders (15). A previous study has shown that human and monkey Doppel are glycosylphosphatidylinositol (GPI)-anchored glycoproteins with N- and O-linked glycosylation (16–18). In this study, we investigated the genetic characteristics and polymorphic landscape of the monkey *PRND* gene. We also examined genotype, allele, and haplotype frequencies and analyzed LD within *PRND* polymorphisms and between *PRNP* and *PRND* variants. In addition, comparative sequence analyses across several prion-related species were performed using ClustalW to identify genetic features intrinsic to monkeys. Furthermore, the potential structural consequences of the identified polymorphisms on the predicted three-dimensional architecture of monkey Doppel were evaluated.

## 2 Materials and methods

### 2.1 Ethical statements and genomic DNA

Blood samples from 121 monkeys were collected by a skilled veterinarian at the Korea Institute of Toxicology following ethical guidelines, and genomic DNA was extracted from 100 μl of blood using the Bead Genomic DNA Prep kit (Biofact, Daejeon, Korea).

### 2.2 Genetic analysis of the monkey *PRND* gene

The cynomolgus monkey (*Macaca fascicularis*) *PRND* gene (Gene ID: 102126856) was amplified from the genomic DNA by polymerase chain reaction (PCR) using gene-specific primers (F: 5′-CCTTCCTTAAAGTCTCCTGC-3′, R: 5′- ATATATGTGGGAGTGGCATTTG-3′). The PCR mixture was composed of 2.5 μl of 10 × *Taq* reaction buffer, 5 μl of 5 × band helper, 1 μl of each 10 mM dNTP mix, 1 μl of each primer (10 μM), 0.2 μl of H-star *Taq* DNA polymerase (BIOFACT, Daejeon, Korea), and sterile water up to a total volume of 25 μl. The PCR was performed with the following experimental conditions: 95 °C for 20 min for denaturation; 34 cycles of 95 °C for 20 s, 58 °C for 40 s, and 72 °C for 1 min for annealing and extension; and 1 cycle of 72 °C for 5 min for the final extension. PCR was performed using a C1000 Touch Thermal Cycler (Bio-Rad, Hercules, California, USA). The PCR products were analyzed by an ABI 3730 sequencer (ABI, Foster City, CA, USA). The sequencing peaks were visualized by FinchTV software (Geospiza Inc., Seattle, WA, USA), and genotyping was performed.

### 2.3 Multiple sequence alignments and phylogenetic tree

The amino acid sequences of *PRND* were obtained from GenBank at the National Center for Biotechnology Information (NCBI), including those of humans (*Homo sapiens*, NP_036541.2), cattle (*Bos taurus*, NP_776583.1), sheep (*Ovis aries*, NP_001009261.1), goats (*Capra hircus*, XP_005688215.2), cats (*Felis catus*, XP_006929990.1), horses (*Equus caballus*, NP_001085006.1), dogs (*Canis lupus familiaris*, XP_038289147.1), rabbits (*Oryctolagus cuniculus*, XP_008254493.2), and monkeys (In this study). The amino acid sequences of *PRND* were subsequently aligned using ClustalW with the progressive alignment method. A phylogenetic tree was constructed using MEGA 12 with the neighbor-joining method (19).

### 2.4 Evaluation of the effect of non-synonymous SNPs by *in silico* analyses

To assess the potential impact of non-synonymous SNPs in monkey Doppel, we employed a suite of computational prediction tools, including SIFT, MutPred2, PolyPhen-2, and AMYCO. To systematically evaluate the functional consequences of amino acid substitutions, we employed several computational approaches. SIFT predicts whether a given substitution is likely to impair protein function, thereby enabling prioritization of variants for downstream investigation. SIFT scores ≤ 0.05 are considered deleterious (20). MutPred2, a machine-learning–based web server, classifies mutations as either disease-associated or neutral, while also estimating their pathogenic potential and underlying molecular mechanisms. A MutPred2 score > 0.5 was considered indicative of potential pathogenicity (21). We used AMYCO, combining PAPA and pWALTZ scores, to evaluate how non-synonymous SNP alleles affect the amyloidogenic potential of monkey Doppel prion-like domains (PrLDs). An AMYCO score below 0.45 indicates low amyloid propensity (22). Among these, PolyPhen-2 infers the functional consequences of amino acid substitutions based on position-specific independent counts (PSIC), classifying variants into two categories of predicted functional alteration: “benign (0.0–0.15)” and “possibly damaging (0.15–1.0)” (23). Because these algorithms assess distinct biological properties-evolutionary conservation, predicted molecular pathogenicity, amyloidogenic propensity, and sequence/structural context-their predictions are not necessarily concordant.

### 2.5 2D and 3D structures prediction and validation of monkey Doppel

Structural information for monkey Doppel was obtained from the predicted protein sequence corresponding to the cynomolgus monkey *PRND* gene (Gene ID: 102126856) for comparative analysis. We used two programs, PSIPRED and GOR IV, to predict the secondary structure of Doppel in humans and monkey. PSIPRED predicts the structure based on position-specific scoring matrices (24). GOR IV predicts protein secondary structure based on information theory and Bayesian statistics (25).

Next, we used Robetta (https://robetta.bakerlab.org/) to compare the 3D structures of wild-type monkey Doppel and mutant Doppel proteins carrying non-synonymous SNPs. Robetta is a web service that offers automated tools for predicting and analyzing protein structures from amino acid sequences (26). Model confidence metrics and backbone root-mean-square deviation (RMSD) values relative to wild-type Doppel were used to assess the overall structural similarity of the predicted models. In addition, Ramachandran plot analysis was conducted by calculating ϕ/ψ dihedral angles to assess and compare the conformational quality and structural stability of wild-type and mutant models based on residues in favored, allowed, and outlier regions.

### 2.6 Statistical analyses

Genotype, allele, and haplotype frequencies, as well as linkage disequilibrium (LD), were analyzed using Haploview (v4.2) (Broad Institute, Cambridge, MA, United States) and SAS/STAT software (SAS Institute Inc., Cary, NC, USA). The Hardy-Weinberg equilibrium (HWE) test was calculated by the chi-square test. A *P*-value < 0.05 was initially considered indicative of deviation from HWE. To account for multiple testing across the 15 *PRND* polymorphisms, Bonferroni correction was applied, resulting in an adjusted significance threshold of *P* < 0.0033 (0.05/15). Such deviations can result from factors including mutation, natural selection, population stratification, or genotyping errors.

## 3 Results

### 3.1 Amplification of the *PRND* gene in monkeys

We designed primers based on the monkey *PRND* gene sequence (Gene ID: 102126856) to obtain an amplicon of the monkey *PRND* gene encompassing the entire ORF. The alignment of the identified monkey *PRND* gene sequence with the reference monkey *PRND* gene sequence registered in GenBank revealed 100% sequence identity.

### 3.2 Identification of polymorphisms in the monkey *PRND* gene

We performed genotyping using amplicon sequencing data of the *PRND* gene in cynomolgus monkeys (*Macaca fascicularis*) and identified 15 novel genetic polymorphisms: c.22C>T, c.61C>T, c.78C>G, c.126T>C, c.129T>C, c.144G>A, c.147C>T, c.255C>T, c.318C>T, c.324T>C, c.370G>C, and c.511G>A in the ORF region and c.-6G>T, c.^*^12G>A, and c.^*^40C>T in the upstream and downstream regions (Figure 1; Table 1). Among the SNPs, c.22C>T (W8R), c.61C>T (L21F), c.370G>C (D124H), and c.511G>A (V171I) were non-synonymous SNPs. The genotype and allele frequencies of the 15 polymorphisms are described in Table 1. We also carried out two LD analyses among the 15 polymorphisms of the monkey *PRND* gene (Table 2) and between *PRNP* and *PRND* polymorphisms (Table 3) by calculating the *r*^2^ values. Among them, c.-6G>T showed strong LD (*r*^2^ > 0.333) with c.126T>C, and c.78C>G showed strong LD with c.511G>A and c.^*^12G>A. In addition, c.144G>A was in perfect LD (*r*^2^ = 1.0) with c.324T>C, and c.511G>A was in perfect LD with c.^*^12G>A. In Table 3, strong LD (*r*^2^ > 0.333) was not observed between monkey *PRNP* and *PRND* polymorphisms. Next, we performed a haplotype analysis of 15 polymorphisms of the monkey *PRND* gene and found ten haplotypes of monkey *PRND* polymorphisms (Table 4). Among them, three major haplotypes were observed at frequencies greater than 10%: GTCCCTGCCCTGGGC (43.4%), TTCCTTGCCCTGGGC (16.5%), and GCCCTTGCCCTGGGC (13.8%).

**Figure 1:**
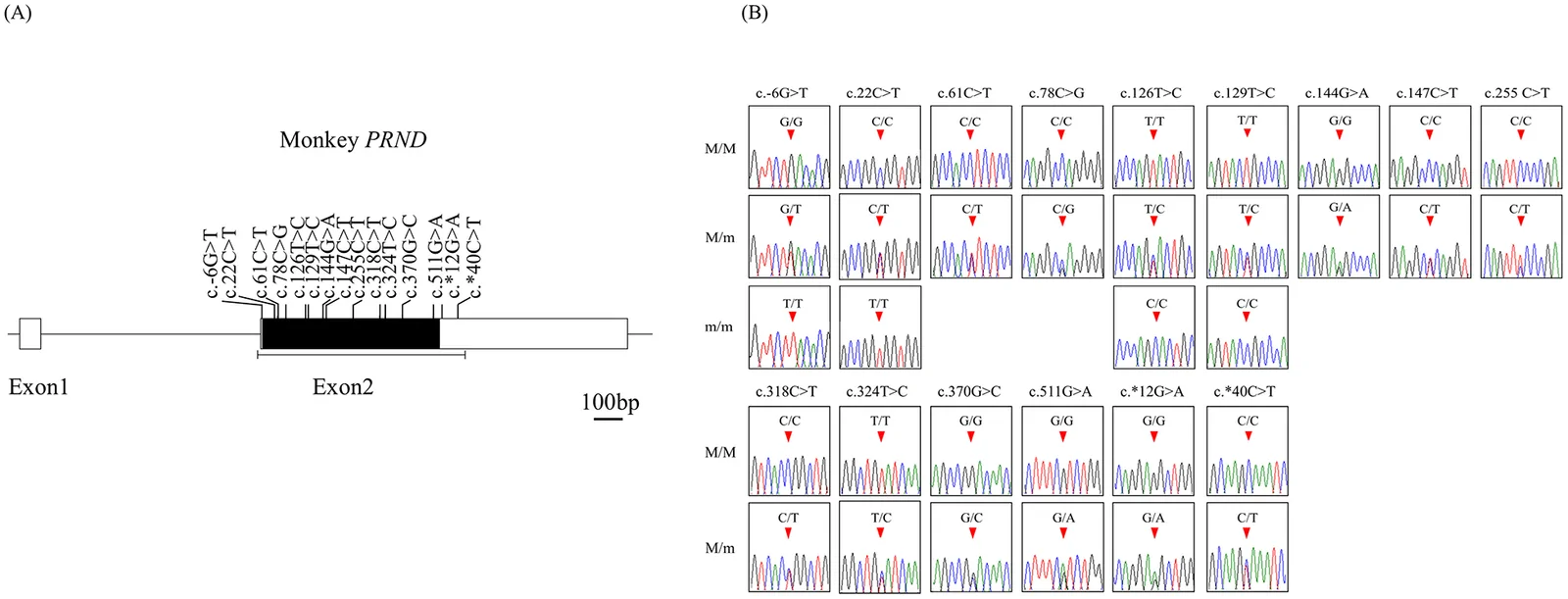
Identification of novel genetic polymorphisms in the monkey prion-like protein gene (*PRND*). **(A)** Gene map and genetic polymorphisms identified in the monkey *PRND* gene. The open reading frame (ORF) within exon 2 is indicated by a shaded black block, and horizontal bars with edges indicate the regions sequenced. The vertical and folded lines indicate the SNPs found in this study. **(B)** Electropherograms of 15 novel single-nucleotide polymorphisms (SNPs): c.-6G>T, c.22C>T, c.61C>T, c.78C>G, c.126T>C, c.129T>C, c.144G>A, c.147C>T, c.255C>T, c.318C>T, c.324T>C, c.370G>C, c.511G>A, c.*12G>A, and c.*40C>T. The colors of the peaks indicate each base of the DNA sequence (green: adenine; red: thymine; blue: cytosine; black: guanine). The red arrows indicate the location of SNPs found in the present study. M/M, M/m, and m/m indicate major homozygotes, heterozygotes, and minor homozygotes, respectively.

**Table 1:** Genotype and allele frequencies of prion-like protein (*PRND*) gene polymorphisms in 121 monkeys.

Polymorphisms	Genotype frequencies, *n* (%)			Allele frequencies, *n* (%)		HWE
	MM	Mm	mm	*M*	*m*	
c.-6G>T	58 (47.93)	50 (41.32)	13 (10.74)	166 (68.60)	76 (31.40)	0.6527
c.22C>T (W8R)	78 (64.46)	39 (32.23)	4 (3.31)	195 (80.58)	47 (19.42)	0.7431
c.61C>T (L21F)	120 (99.17)	1 (0.83)	0 (0.0)	241 (99.59)	1 (0.41)	0.9635
c.78C>G (A26A)	120 (99.17)	1 (0.83)	0 (0.0)	241 (99.59)	1 (0.41)	0.9635
c.126T>C (S42S)	39 (32.23)	57 (47.11)	25 (20.66)	135 (55.79)	107 (44.21)	0.6200
c.129T>C (T43T)	113 (93.39)	7 (5.79)	1 (0.83)	233 (96.28)	9 (3.72)	0.0345
c.144G>A (E48E)	114 (94.21)	7 (5.79)	0 (0.0)	235 (97.11)	7 (2.89)	0.7431
c.147C>T (A49A)	120 (99.17)	1 (0.83)	0 (0.0)	241 (99.59)	1 (0.41)	0.9635
c.255C>T (F85F)	118 (97.52)	3 (2.48)	0 (0.0)	239 (98.76)	3 (1.24)	0.8901
c.318C>T (T106T)	105 (86.78)	16 (13.22)	0 (0.0)	226 (93.39)	16 (6.61)	0.4361
c.324T>C (C108C)	114 (94.21)	7 (5.79)	0 (0.0)	235 (97.11)	7 (2.89)	0.7631
c.370G>C (D124H)	120 (99.17)	1 (0.83)	0 (0.0)	241 (99.59)	1 (0.41)	0.9635
c.511G>A (V171I)	119 (98.35)	2 (1.65)	0 (0.0)	240 (99.17)	2 (0.83)	0.9269
c.^*^12G>A	119 (98.35)	2 (1.65)	0 (0.0)	240 (99.17)	2 (0.83)	0.9269
c.^*^40C>T	106 (87.60)	15 (12.40)	0 (0.0)	227 (93.80)	15 (6.20)	0.4673

MM, Major homozygote; Mm, Heterozygote; mm, Minor homozygote; HWE, Hardy-Weinberg equilibrium.

**Table 2:** Linkage disequilibrium (LD) among *PRND* polymorphisms in 121 monkeys.

Polymorphisms	c.-6 G>T	c.22 C>T	c.61 C>T	c.78 C>G	c.126 T>C	c.129 T>C	c.144 G>A	c.147 C>T	c.255 C>T	c.318 C>T	c.324 T>C	c.370 G>C	c.511 G>A	c.^*^12 G>A	c.^*^40 C>T
c.-6G>T	–														
c.22C>T	0.082	–													
c.61C>T	0.009	0.017	–												
c.78C>G	0.009	0.001	0.0	–											
c.126T>C	**0.363**	0.161	0.003	0.003	–										
c.129T>C	0.032	0.009	0.0	0.0	0.031	–									
c.144G>A	0.065	0.007	0.0	0.0	0.024	0.004	–								
c.147C>T	0.009	0.017	0.0	0.0	0.003	0.0	0.0	–							
c.255C>T	0.027	0.003	0.0	0.0	0.01	0.026	0.0	0.0	–						
c.318C>T	0.032	0.021	0.0	0.059	0.056	0.006	0.002	0.0	0.177	–					
c.324T>C	0.065	0.007	0.0	0.0	0.024	0.004	**1.0**	0.0	0.0	0.002	–				
c.370G>C	0.002	0.001	0.0	0.0	0.005	0.0	0.0	0.0	0.0	0.0	0.0	–			
c.511G>A	0.018	0.002	0.0	**0.498**	0.007	0.0	0.0	0.0	0.159	0.118	0.0	0.0	–		
c.^*^12G >A	0.018	0.002	0.0	**0.498**	0.007	0.0	0.0	0.0	0.159	0.118	0.0	0.0	**1.0**	–	
c.^*^40C>T	0.155	0.017	0.0	0.0	0.056	0.003	0.002	0.0	0.001	0.005	0.002	0.0	0.001	0.001	–

Bold text indicates strong LD (*r*^2^ > 0.333).

**Table 3:** Linkage disequilibrium (LD) analysis between polymorphisms of *PRNP* and *PRND* genes with r^2^ value in monkeys.

Polymorphisms	c.-6 G>T	c.22 C>T	c.61 C>T	c.78 C>G	c.12 6T>C	c.129 T>C	c.144 G>A	c.147 C>T	c.255 C>T	c.318 C>T	c.324 T>C	c.370 G>C	c.511 G>A	c.^*^12 G>A	c.^*^40 C>T
c.117G>A	0.014	0.001	0.003	0.006	0.035	0.003	0.0	0.006	0.018	0.003	0.0	0.006	0.012	0.012	0.0
c.138C>T	0.012	0.001	0.0	0.0	0.011	0.001	0.001	0.0	0.0	0.001	0.001	0.0	0.0	0.0	0.001
c.213C>T	0.010	0.005	0.0	0.0	0.006	0.001	0.001	0.0	0.0	0.001	0.001	0.0	0.0	0.0	0.002
c.226_249del CCTCATGGTGGCGGCTGGGGACAG	0.002	0.0	0.0	0.0	0.0	0.001	0.001	0.197	0.0	0.001	0.001	0.0	0.0	0.0	0.002
c.237C>T	0.039	0.0	0.0	0.0	0.01	0.106	0.002	0.073	0.014	0.004	0.002	0.0	0.0	0.0	0.036
c.239G>C	0.018	0.002	0.0	0.0	0.011	0.046	0.0	0.0	0.0	0.021	0.0	0.0	0.0	0.0	0.021
c.323G>A	0.009	0.017	0.0	0.0	0.003	0.0	0.0	0.0	0.0	0.0	0.0	0.0	0.0	0.0	0.0
c.378C>A	0.002	0.004	0.0	0.0	0.0	0.007	0.001	0.0	0.0	0.002	0.001	0.0	0.0	0.0	0.002
c.537C>T	0.002	0.0	0.003	0.006	0.027	0.004	0.019	0.006	0.019	0.014	0.019	0.006	0.013	0.013	0.002
c.576C>T	0.010	0.005	0.002	0.008	0.023	0.002	0.016	0.008	0.023	0.0	0.016	0.008	0.015	0.015	0.002

The vertical and horizontal axes represent *PRNP* and *PRND* polymorphisms, respectively. The *PRNP* genotyping data used for this analysis were previously generated and validated by our research group using the same cohort of 121 cynomolgus monkeys; the corresponding *PRNP* manuscript is currently under revision.

**Table 4:** Haplotype frequencies of 15 *PRND* polymorphisms in monkeys.

Haplotype	Frequency (*n* = 242)
GTCCCTGCCCTGGGC	105 (0.434)
TTCCTTGCCCTGGGC	40 (0.165)
GCCCTTGCCCTGGGC	33 (0.138)
TTCCTTGCCCTGGGT	16 (0.066)
GTCCTTGCCCTGGGC	14 (0.059)
GCCCTTGCCTTGGGC	7 (0.029)
TTCCTTACCCCGGGC	7 (0.027)
TTCCTTGCCTTGGGC	4 (0.016)
TTCCTCGCCCTGGGC	3 (0.013)
GCCCTCGCCCTGGGC	2 (0.010)
Others	11 (0.043)

Others contain rare haplotypes with frequency < 0.01.

### 3.3 Multiple sequence alignments of Doppel among various species

We performed multiple sequence alignments of Doppel amino acid sequences among humans, cattle, sheep, goats, cats, horses, dogs, rabbits, and monkeys (Figure 2A). Humans and monkeys showed the same sequence length (176 aa). The expected interaction regions between Doppel (residues 80–120; red box) and the N-terminal domain of PrP (residues 23–90) are shown. The mature ectodomain of Doppel adopts an α-helix–rich globular fold and includes two conserved N-linked glycosylation motifs located at N98 and N110 in Doppel (black boxes), which are conserved among all species. Sequence homology is extremely high in the N-terminal and C-terminal regions among mammals. Signal peptide (purple boxes) and GPI anchor (green boxes) analyses of the amino acid sequences of *PRND* are also presented. The phylogenetic tree indicated that monkeys and humans clustered within the same branch (Figure 2B).

**Figure 2:**
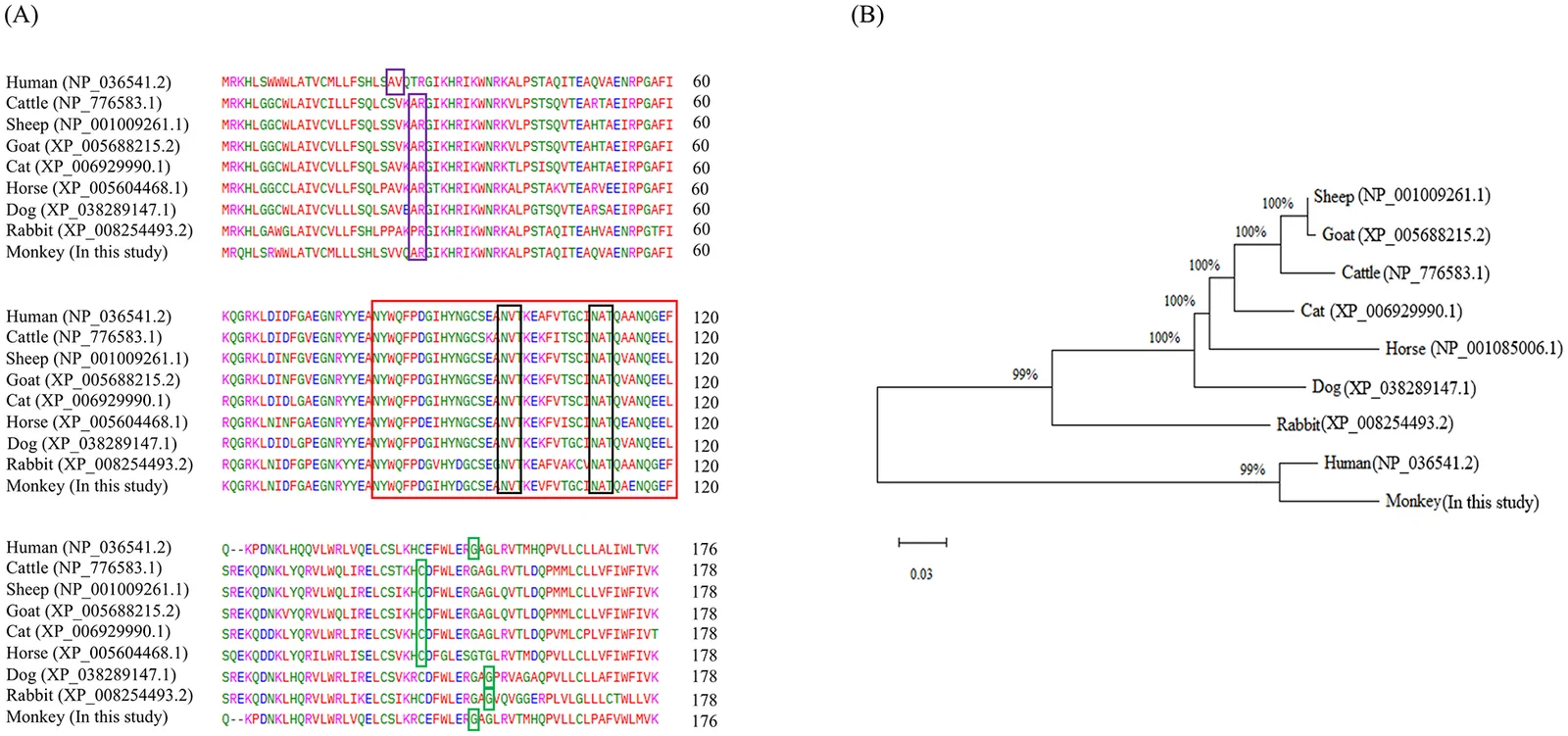
**(A)** Multiple sequence alignments of amino acid sequences of the prion-like protein (Doppel) in humans, cattle, sheep, goats, cats, horses, dogs, rabbits, and monkeys. Amino acids are color-coded based on their chemical properties: acidic residues in blue, hydrophobic in red, basic in magenta, and hydroxyl, sulfhydryl, amine, and glycine in green. Purple boxes indicate the signal peptide of Doppel. The red box indicates the predicted interaction region between Doppel and PrP. Black boxes indicate N-linked glycosylation motifs. Green boxes indicate the GPI anchor. **(B)** Phylogenetic tree of Doppel amino acid sequences among various species.

### 3.4 Comparison of the *PRND* gene polymorphisms across various species

We conducted a comparative analysis of genetic variation distributions in the *PRND* ORF region across different species (Figure 3). Among the species examined, monkeys exhibited the highest number of polymorphisms in the *PRND* gene.

**Figure 3:**
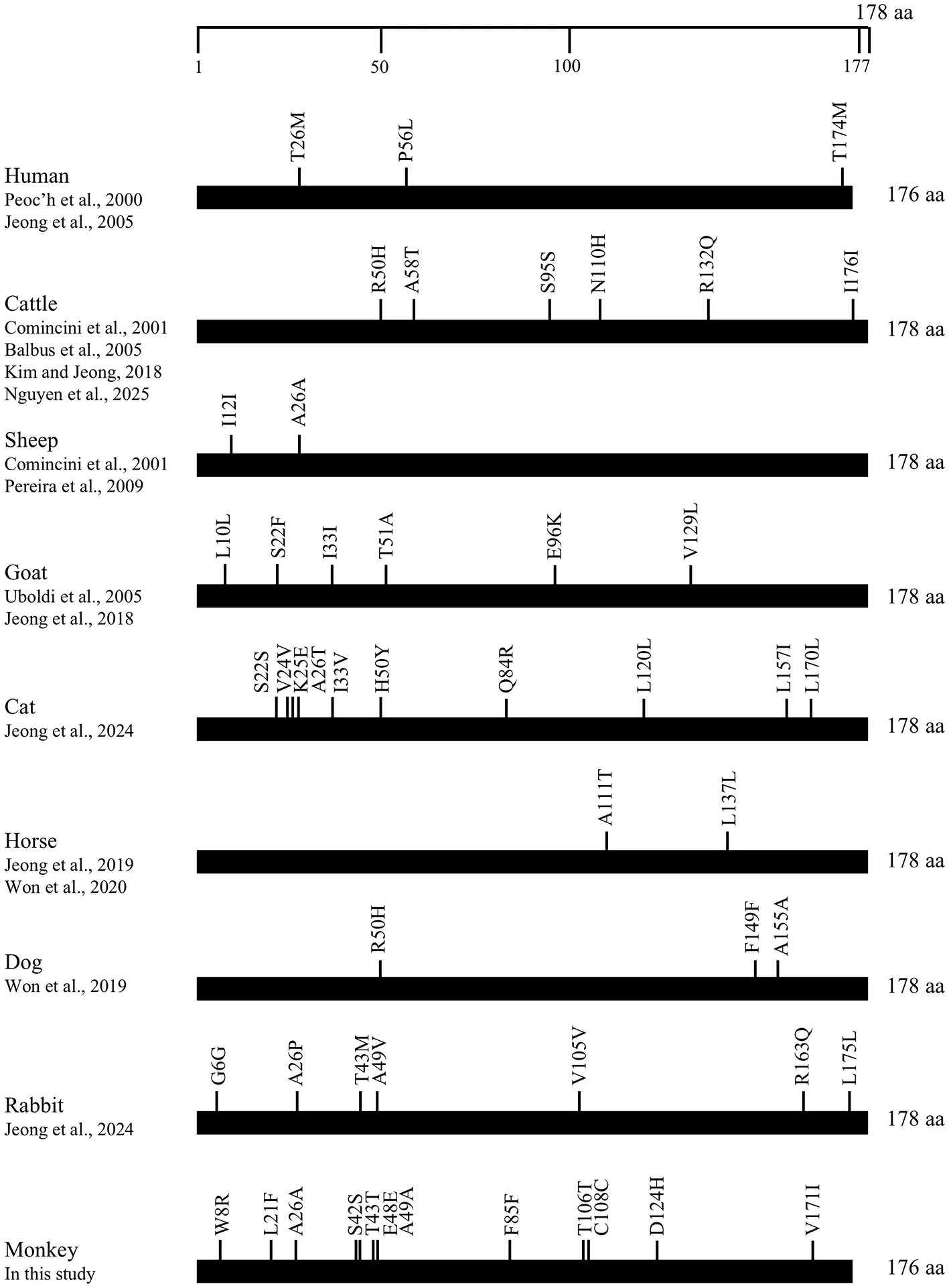
Distribution of genetic polymorphisms in the open reading frame (ORF) of the *PRND* gene in various species. The figure shows the previously reported genetic polymorphisms of the *PRND* gene in humans (27, 39), cattle (40–43), sheep (40, 44), goats (14, 45), cats (46), horses (29, 47), dogs (30), rabbits (48), and monkeys. The edged horizontal bar indicates the amino acid length of Doppel.

### 3.5 *In silico* evaluation of the effects of non-synonymous SNPs

To investigate the influence of genetic polymorphisms on monkey Doppel, we used SIFT, MutPred2, AMYCO, and PolyPhen-2 (Table 5). The results showed that the non-synonymous SNPs c.61C>T (L21F) and c.370G>C (D124H) were predicted to be “deleterious” by SIFT and “possibly damaging” by PolyPhen-2. In contrast, c.22C>T (W8R) and c.511G>A (V171I) were predicted to be “tolerated” by SIFT, with scores of 0.19 and 1.0, respectively. MutPred2 and AMYCO did not predict marked pathogenicity or increased amyloid propensity for these variants.

**Table 5:** *In silico* evaluation of the effects of polymorphisms on the *PRND* gene in monkeys.

Polymorphisms	Method	Score	Prediction
c.22C>T (W8R)	SIFT	0.19	Tolerance
	MutPred2	0.304	Benign
	AMYCO	0.0	Neutral
	PolyPhen-2	0.079	Benign
c.61C>T (L21F)	SIFT	0.0	Deleterious
	MutPred2	0.299	Benign
	AMYCO	0.0	Neutral
	PolyPhen-2	0.675	Possibly damaging
c.370G>C (D124H)	SIFT	0.01	Deleterious
	MutPred2	0.278	Benign
	AMYCO	0.0	Neutral
	PolyPhen-2	1.000	Possibly damaging
c.511G>A (V171I)	SIFT	1.0	Tolerance
	MutPred2	0.045	Benign
	AMYCO	0.0	Neutral
	PolyPhen-2	0.000	Benign

### 3.6 Prediction of secondary structures of human and monkey Doppel

We used two programs, PSIPRED and GOR IV, to predict the secondary structure of Doppel in humans and monkeys (Figure 4). PSIPRED analysis revealed α-helical contents of 48.29% and 47.72% in human and monkey Doppel, respectively (Figures 4A, B). Next, secondary structure analysis using the GOR IV server revealed that the human Doppel protein was comprised of 26.14% α-helices (46 residues), 49.43% random coils (87 residues), and 24.43% extended strands (43 residues) (Figure 4C). For monkey Doppel, GOR IV analysis showed 28.41% α-helices (50 residues), 48.3% random coils (85 residues), and 23.3% extended strands (41 residues) (Figure 4D).

**Figure 4:**
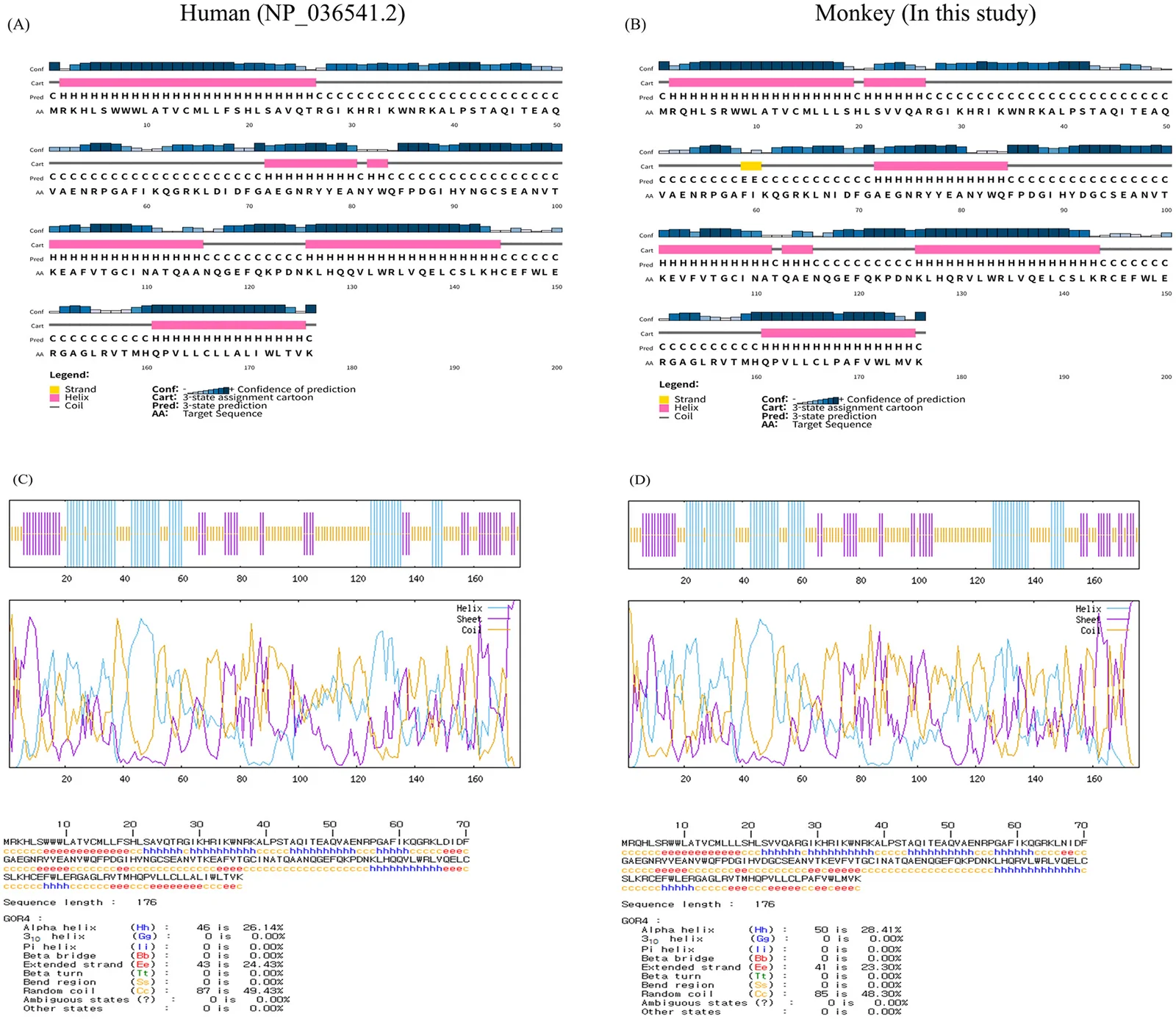
Secondary structure prediction of the Doppel using PSIPRED and GOR IV. **(A)** Secondary structure of human Doppel predicted by PSIPRED. **(B)** Secondary structure of monkey Doppel predicted by PSIPRED. The colors correspond to the secondary structure elements as shown in the legend. The letters C, H, and E denote coil, helix, and strand regions, respectively. **(C)** Secondary structure of human Doppel predicted by GOR IV. **(D)** Secondary structure of monkey Doppel predicted by GOR IV.

### 3.7 Comparison of the 3D structures between wild-type and mutant monkey Doppel

Subsequently, we utilized Robetta to predict the 3D structures of wild-type monkey Doppel and a double-mutant Doppel model carrying both L21F and D124H substitutions (Figure 5A). The predicted mutant structures showed low backbone RMSD values (< 0.45 Å) relative to the wild-type structure, indicating overall conservation of the tertiary fold. Local structural analysis of the double-mutant model suggested minor side-chain packing changes around the L21F substitution and a modest alteration in local electrostatic properties around the D124H substitution, while the overall α-helical backbone geometry was preserved.

**Figure 5:**
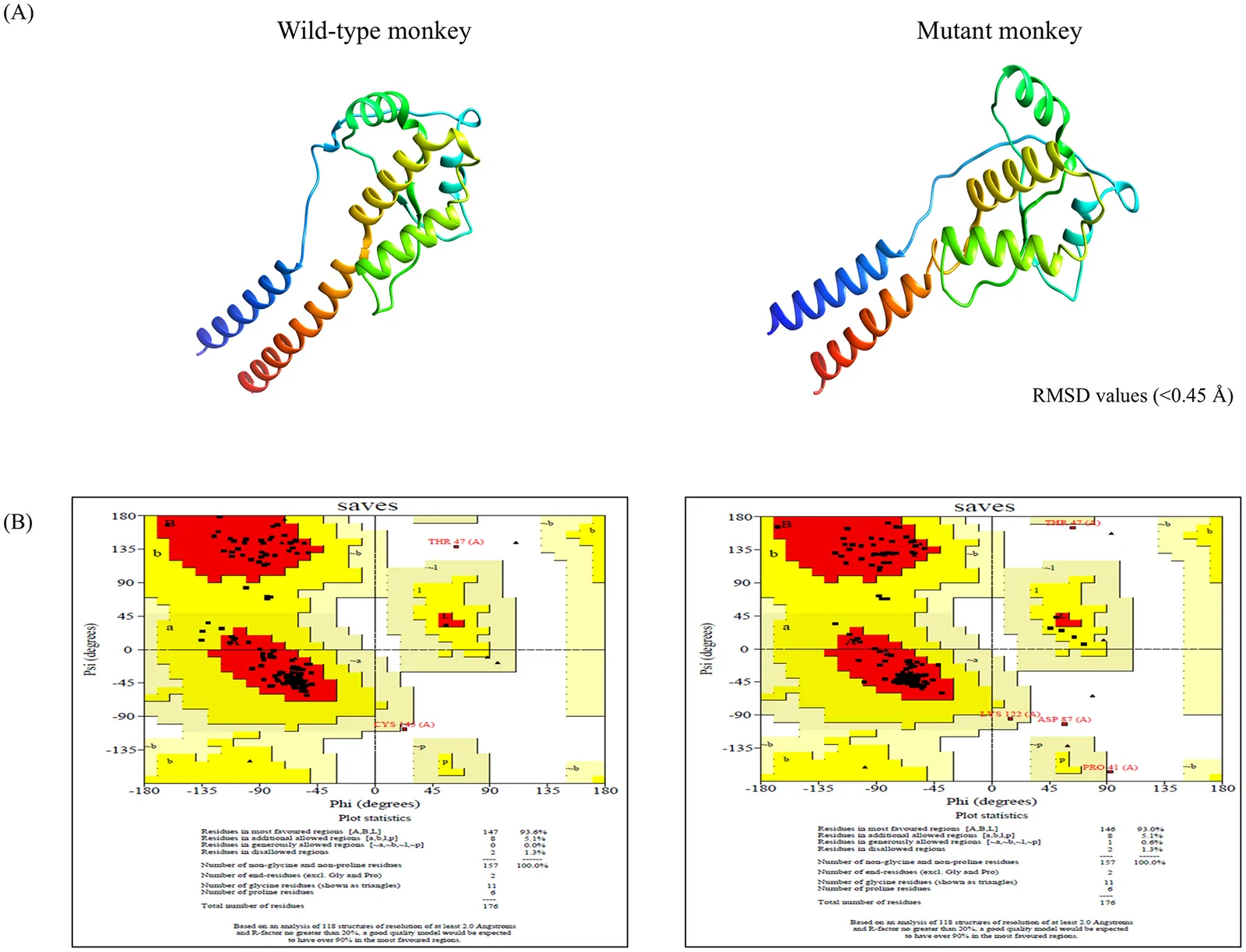
3D protein structures prediction of the Doppel based on the Robetta algorithm, the *de novo* modeling of three-dimensional protein structures directly from amino acid sequences. **(A)** The 3D models of the Doppel were obtained on the Robetta server. The left panel shows the wild-type monkey, and the right panel shows the mutant monkey. **(B)** Ramachandran plot analysis of protein structures. The left panel shows the wild-type monkey, and the right panel shows the mutant monkey. This analysis evaluates the validity of the protein models by assessing the dihedral angles, confirming the similarity between the two models.

Structural consistency between the wild-type and mutant models was further supported by Ramachandran plot analysis (Figure 5B). The overall structural integrity was highly conserved, with over 93% of residues in favored regions. However, local backbone rearrangements were observed in the double-mutant model, including changes involving proline 41, threonine 47, aspartic acid 87, lysine 122, and cysteine 145. These alterations were confined to specific loops or turns and did not substantially affect the global fold of Doppel.

## 4 Discussion

The monkey *PRND* gene showed 94.35% sequence identity with the human *PRND* gene (Gene ID: 23627), and amino acid substitutions previously associated with prion susceptibility in humans were not detected in monkeys. Hence, we investigated *PRND* polymorphisms in monkeys. In the present study, we identified 15 novel SNPs, including four non-synonymous SNPs (Figure 1; Table 1). One limitation of this study is the relatively small cohort size. Several SNPs were observed in only a single heterozygous individual among the 121 monkeys, which may limit the robustness of allele-frequency and haplotype analyses for these rare variants.

In humans, *PRND* polymorphisms have been reported, comprising three amino acid substitutions (T26M, P56L, and T174M) (27). Especially, T174M has been suggested to be associated with prion disease susceptibility or progression (28). None of the SNPs found in monkeys overlapped with SNPs associated with prion disease in humans. Next, to explore the potential genetic relationship between *PRNP* and *PRND*, we performed a linkage disequilibrium (LD) analysis (Table 3). Consistent with findings in horses (29) and dogs (30), monkeys exhibited weak LD between polymorphisms in these two loci. In contrast, black goats demonstrated a pronounced genetic linkage between *PRNP* and *PRND* (14). Although naturally occurring prion infections have not been documented in monkeys, they remain a highly relevant model system in prion biology (31, 32). Continued investigation is warranted to elucidate the *PRND* genetic diversity and its implications for prion susceptibility in primates. Previous structural analyses of human and monkey PrP have provided a framework for interpreting the physiological implications of *PRNP* polymorphisms in monkeys. We also predicted the secondary structures of human and monkey Doppel in this study (Figure 4). Notably, the high proportion of residues predicted as random coils (48.3–49.43%) by GOR IV suggests substantial non-regular secondary structure, particularly in inter-helical loops and terminal regions (33). Next, two non-synonymous SNPs, c.61C>T (L21F) and c.370G>C (D124H), were predicted to be possibly damaging by *in silico* tools (Table 5). However, there was no significant difference between the wild-type monkeys and mutant monkeys carrying these SNPs in the predicted 3D structures (Figure 5A). This apparent discrepancy may reflect differences between sequence-based functional prediction algorithms and static 3D structure prediction, as subtle or transient conformational effects of amino acid substitutions may not be readily captured by a single predicted structure (26). Ramachandran plot analysis was further used to assess the stereochemical quality of the predicted structures (Figure 5B). Since static 3D structure prediction alone cannot fully evaluate protein conformational changes, it is important to perform molecular dynamics (MD) simulations as well as experimental validations *in vivo* or in cell lines to assess how these SNPs affect protein function and interactions.

Recent studies have identified Doppel as a biomarker for ovarian cancer (34) and demonstrated its role in aiding recovery after ischemic stroke (35). When overexpressed in neurons, Doppel can induce cytotoxic effects, including neuronal cell death, whereas PrP is known to confer protection against such toxicity (36). In *PRNP*-deficient mice, upregulation of *PRND* is associated with the development of cerebellar ataxia, supporting a potential neurotoxic role for Doppel. Importantly, genetic ablation of *PRND* in a *PRNP*-null background fully rescues the neurodegenerative phenotype, thereby establishing that the observed neurotoxicity arises not from the absence of *PRNP* itself but from ectopic expression of Doppel. Monkeys are frequently used as models for cytotoxicity assessment and therapeutic development (37). Although further research is needed regarding Doppel-induced cytotoxicity and cell death, monkeys may serve as a valuable model for investigating these mechanisms. Furthermore, recent studies examining prion-like sequence features in coronaviruses illustrate the broader application of computational approaches for evaluating prion-like domains and sequence-dependent structural propensity (38).

## 5 Conclusions

In conclusion, we identified 15 genetic polymorphisms in the *PRND* gene of cynomolgus monkeys (*Macaca fascicularis*), including several strong LD relationships and three major haplotypes. *In silico* predictions identified two non-synonymous variants, c.61C>T (L21F) and c.370G>C (D124H), as potentially damaging. While secondary and 3D structural predictions showed overall structural conservation between wild-type and mutant monkey Doppel proteins, future research incorporating long-term molecular dynamics (MD) simulations and *in vitro* functional assays will be critical to determine the dynamic effects of these novel variants on protein stability, cell surface expression, and neurotoxicity. Ultimately, these baseline findings advance our understanding of primate *PRND* genetic diversity and provide a useful foundation for translational studies on host susceptibility to prion and neurodegenerative disorders.

## Data Availability

Data are available from the corresponding author upon reasonable request. The nucleotide sequence data generated in this study have been deposited in GenBank under accession number PZ526972.
